# Exploring the Influence of the Community-Based Sports Club Environment on the Support and Development of Volunteer Women Coaches in Ireland

**DOI:** 10.3389/fspor.2022.809092

**Published:** 2022-03-31

**Authors:** Irene Hogan, Richard Bowles, Niamh Kitching

**Affiliations:** Mary Immaculate College, Limerick, Ireland

**Keywords:** volunteer, women, coaching, experiences, ladies Gaelic football, community sports club

## Abstract

In Ireland, the majority of coaches at non-elite level are volunteers and within the female-only team sport of women's Gaelic football, most qualified coaches are women. Yet, little is known on the club specific experiences of volunteer women coaches in non-elite sport. To address this gap, 11 women coaches, from three Gaelic Football clubs, were interviewed to explore the influence of the community-based club environment on their support and development in the role. The participants were actively coaching and part of a Community of Practice (CoP) focusing on developing their club's coaching structures. A creative non-fiction approach combined the key themes from the 11 interviews into three coach profiles of a novice coach, experienced coach, and a player-coach. Retention and recruitment, support structures within the club, and club culture and norms were the key themes identified. This study recommends that clubs employ support structures that support and develop volunteer women coaches and address any behavior in the club that negatively impacts on their role.

## Introduction

Internationally, the majority of coaches are at non-elite voluntary level (LaVoi, 2016) and are considered the cornerstone of sports organizations (Walsh, 2015), yet continue to be under researched (Griffiths and Armour, 2012; Trussell, 2016; Wicker, 2017). In the UK, most (89%) are at recreational and club level, with almost half (46%) of all coaches doing so in a voluntary capacity (UK Sports Coach, 2019). Although a coaching workforce survey has not yet been conducted in Ireland, some of the National Governing Bodies (NGB's) have completed their own sport specific version. In line with previous research, among 10, 647 Gaelic games coaches surveyed, 90% of the respondents coached at club level. Further, only 20% of the respondents were female (Horgan et al., 2021). This is in line with the EU Gender Quality in Sport Report 2014 that highlighted 20–30% of all coaches in Europe are female with figures varying between countries and sports (European Commission, 2014). Hence, governing bodies should organize campaigns to recruit more women coaches, whilst also putting structures in place to monitor their development, education, and retention (European Commission, 2014).

Volunteer coaches have a complex set of relationships with parents and committee members and often work in a vacuum with little research evidence available to inform their practice (Cronin and Armour, 2015). Research to date on volunteer coaches has been centered around their motivations to start coaching but less is known about their experiences once involved. One concern previously reported by sports clubs is that once a volunteer coach has commenced their role, their engagement proves more difficult to predict (Egli et al., 2014). Consequently, more research is needed in order to explore the experiences of volunteer coaches (McNeill et al., 2017) from a variety of contexts in their respective coaching environments. Volunteer coaching is multi-faceted, with increasing workload and expectations from parents and club members alike (Hoye et al., 2019) and arguably for women, it is even more complex. Internationally, volunteer women coaches are behind their male counterparts and underrepresented in coaching research causing a knowledge gap for this group of coaches (Pfister and Norman, 2017). Potts et al. (2019) call for research on women coaches at different levels of coaching as well as at different transitional points in their lives. Burton and LaVoi (2016) go further and believe research needs to understand the organizational and socio-cultural challenges that are also impacting women coaches. Yet, little is known about volunteer, non-elite women coaches' experiences, particularly at grassroots level in community-based sports clubs in Ireland.

Currently, around 400,000 volunteer coaches in Ireland contribute on average 3.5 h per week to coaching and only 32% of these are women (Sport Ireland, 2021). The National Sports Policy 2018–2027 (Government of Ireland, 2018) wants to increase this figure but to achieve such an increase, it is important to understand how to attract, develop and retain women coaches. Similar to Schlesinger et al. (2021) findings, Ireland does not have any outward exclusions, rules, or procedures that prevent women from coaching or attending coach education. Yet, it is unknown what hidden and/or informal challenges they face when they do engage in these activities. Such latent obstacles might include gender bias, a lack of role models, low confidence, and limited support structures. Although Norman and Simpson (2022) focused on elite women coaches the micro-aggressions they referred to, such as sexism and micro-invalidations, could also be the case for volunteer women coaches who work in the male dominated coaching environment. Moreover, women coaches are often faced with additional complexities to start and continue in the role as many work full time, possibly alongside extra responsibilities at home. There is a 40% and 14% gap between men and women in Ireland for household duties and caring responsibilities, respectively [European Institute for Gender Equality (EIGE), 2020]. Further challenges include the gender bias and stereotypes that impact the recruitment, retention, and development of women coaches at all levels (Burton and Newton, 2021). It is imperative to examine these challenges and why they occur (Hovden and Tjønndal, 2021) as women tend to quit volunteering quicker than their male counterparts, resulting in a continuous cycle of low numbers of women coaches (Burton and Newton, 2021). Hence, we must examine their club specific experiences and make recommendations to those in decision making positions, such as sports' NGB's.

The NGB at the center of this study is the Ladies Gaelic Football Association (LGFA), governing the amateur sport of Ladies Gaelic football, with over 188,000 members across 1,200 clubs (LGFA, 2018). The LGFA's remit is to organize non-elite club and elite inter-county competitions in Ireland, and in some international communities. The LGFA also have a recreational programme, aimed at females over 25 years of age and not playing competitively, known as Gaelic for Mothers and Others (G4M&O). In Ireland, women account for 65% of those that have completed LGFA introductory level coach education (LGFA, 2020), which significantly exceeds the national rate of 37% across other sports (Sport Ireland Coaching, 2019). Given the high numbers of qualified women's Gaelic football coaches, it is essential to understand their experiences within the club environment, so improvements can be made, where necessary.

The LGFA 2017-2022 Strategic Plan (LGFA, 2018) considers volunteers as the foundation of the association and deem it vital to provide for their continuous development; especially with the increased demand and expectations they encounter. The strategy aims to improve the alignment between the NGB at national level and the grassroots club via assessing and auditing of current volunteer structures and, subsequently, enhancing them. Underpinning the strategy is to promote female leadership across the sport, which includes coaching. Such an approach, is not unusual according to Harman and Doherty (2014) given the significant contribution that volunteer coaches provide to sport. Understanding the factors that influence their recruitment and retention is essential to ensuring the long term sustainability of sport; particularly for community level organizations (Hoye et al., 2019).

There is very little known on the impact organizations have on volunteers particularly given the changing nature of volunteer attitudes and expectations, thus there is a requirement for innovative ways of managing volunteers (Hoye and Kappelides, 2021). Furthermore, women in coaching research must include an understanding of gender within organizations (Pape, 2019) as many of these processes rely on socially constructed ideas of masculinity and femininity and can lead to biases and stereotypes for women at all levels of coaching (Burton and Newton, 2021). Rundle-Thiele and Auld (2009) call for research to focus on the push and pull factors for volunteer coaches within an organization. Such factors may include training and support of coaches, which Griffiths and Armour (2012) consider key. The majority of training is organized by NGBs through their sport specific coach education programmes.

NGBs must understand the needs of their volunteer coaches, which center around access to appropriate coaching resources and feeling supported and valued in the role (Nash and Sproule, 2012). Subsequently, any effort to enable them to feel part of something and develop, such as involvement in the design and implementation of coach education, should aid their retention (Nash and Sproule, 2012). In addition, understanding how the club structure can impact long term volunteer commitment (Schlesinger and Weigelt-Schlesinger, 2013) is warranted; alongside acknowledging that time is a stressor for volunteer coaches (Potts et al., 2019). An awareness of the club's influence on volunteer women coaches' experiences would answer Schinke et al. (2020) request for applicable research that makes an original contribution to the sport and exercise domain.

This research builds on previous work by the authors including an applied study on female women's Gaelic football coaches, that used LaVoi (2016) Ecological Intersectional Model (EIM) as a framework to explore their experiences (Hogan et al., 2021). Findings revealed that interpersonal support from family, friends, and fellow coaches was key to their continuation; while club-based, context specific support facilitated their development (Hogan et al., 2021). These findings led to the establishment of a Community of Practice (CoP) with three women's Gaelic football clubs, on an individual basis, to focus on their self-identified goals. Culver et al. (2019) champion this self-directed learning approach as a positive aspect of CoPs and Banwell et al. (2019) favors this method over the one size fits all approach of standardized coach education. The CoP was designed and facilitated by the first author as part of a different project that became linked to and embedded in the earlier phase of this research. The CoP afforded the first author access to women coaches as the interviews and CoP sessions ran concurrently. Eleven women coaches, from the three CoPs, were interviewed to further understand the club-based support and development required by volunteer women coaches and assist the NGB with tools to enhance their club environment.

To best address the aims of this research, the authors considered using a theoretical framework to form the interview guide and aid with analysis and interpretation. Depending on the perspective that the researcher takes, some theories are useful and others may be dismissed (Wicker and Hallmann, 2013). This study adopted a combination of the Psychological Contract (PC) and the Ecological Intersectional Model (EIM) to frame the study. Burton and LaVoi (2016) state that an ecological model can be used to examine women coaches' experiences across the individual, interpersonal, organizational, and socio-cultural levels. The EIM was used as an add on to the previous phase of this research on women coaches' experiences where the interpersonal and individual layers prevailed (Hogan et al., 2021).

This study aims to establish the influence of the club's environment (organizational layer) on the support and development of volunteer women coaches and so the PC was introduced to delve further into this layer of the EIM. The PC covers both transactional (e.g., economic) and relational (e.g., socio-emotional) exchanges between the volunteer and the organization (Taylor et al., 2006). Hoye et al. (2019) promote PC as a lens to view the relationship between the volunteer and the organization, while also considering the multiple variables that impact their experiences and, subsequently, identify ways in which the organization can enhance this relationship. Furthermore, the PC enables the examination of volunteer perceptions regarding the value the organizations place on their contributions (Kappelides et al., 2019). Millar and Doherty (2016) call for organizations to build their capacity for more volunteers but to do so organizations must be aware of their needs and come up with strategies to address any issues. Therefore, this study can address this call and build on the work of Hanlon et al. (2019) to help organizations improve their ability to integrate more women. However, this would be an onerous task without first understanding their organizational level experiences. Hence, both the EIM and the PC were used in the research design, analysis and interpretation which were also influenced by the researcher's background.

## Materials and Methods

### Researcher Background

This research was undertaken by the first author, a white, female Irish citizen, with 27 years playing experience at non-elite level and 21 years as a volunteer coach with club youth, adult and inter-collegiate teams in ladies Gaelic football. Since I finished playing, my involvement in women's Gaelic football has been as a coach, coach developer, or coach developer assessor giving me a unique perspective and understanding of volunteer coaches. I identify the impact of my perspective as a volunteer coach, on participant engagement and the conclusions reached in this study (Yin, 2015). A more in-depth reflection on my women's Gaelic Football roles is ongoing for another research article as part of the overall doctoral research project.

Research involves the intersection of philosophy, research design, and methods, in that the researcher must understand their assumptions on worldviews in order to apply a research design (Creswell and Creswell, 2017). Given my background, knowledge, and experiences, I adopt a constructivist stance, to understand participants' perspectives, by interacting with them and using my background to interpret their experiences (Creswell, 2014). It is important, however, to acknowledge the influence of a researcher's epistemological and ontological positions on the creation of the stories told in research (Smith et al., 2015). I align to interpretivism as a way to view the world, as a social interaction between thoughts, emotions, and behaviors that can be used to understand coaches' experiences and report their reality (Gearity et al., 2016). Linked to my constructivist stance, I adopt an insider position based on my previous and current women's Gaelic football roles, so I can empathize with some of their experiences. Overall, social constructivism and interpretivism guided the research design, interpretation, and writing phases of this study.

### Research Design

Semi-structured interviews were undertaken with 11 coaches, from the three CoP clubs, following ethical approval from the authors' home institute, the Mary Immaculate College Research Ethics Committee (A18-035).

### Participant Recruitment

As part of the wider research project, I designed and facilitated a CoP in three clubs, throughout the 2021 playing season. My involvement focused on supporting their club's coaching pathway and structures through meetings with the club members and more regular phone and email interactions with their appointed co-ordinator. For the duration of this study, including the CoP rollout, COVID-19 restrictions were in place requiring all meetings to be held virtually, via Zoom (Zoom Inc.). For the most part, COVID-19 restrictions aided the attendances at the online CoP as the numbers declined once government restrictions were reduced allowing sport to recommence. However, for some the online environment did not suit and subsequently, these individuals never attended the sessions. Although this study took place during COVID-19, it was not as a result of COVID-19, as it was planned in advance of the pandemic and so it was a contributing factor but not the main variable. For example, I was cognizant that women reported higher levels of perceived stress, compared to males, during the lockdown period in 2020 (Santi et al., 2021).

Criterion sampling was used and women that attended at least one of the CoP sessions, and who were actively coaching for the 2021 playing season were included in the recruitment process (*n* = 12). I emailed four women coaches from each club, and six of the twelve agreed to take part. A further five signed up after a follow up email. The only coach that was contacted but did not take part had not replied to any communications about the research and had also stopped attending the CoP after the first two sessions. As the CoP facilitator, I had an insider role in the respective clubs, through meeting them on eight occasions each throughout the 2021 playing season. Being a member of the sub-group or culture helps to gain access to the participants and understand them (Sparkes and Smith, 2014). The CoP gave context to the interviews to compare my observations throughout the CoP with what the women reported. Observing the interactions between the women coaches and their fellow club members helped form the interview guide and the subsequent analysis. For example, when coaches mentioned their club did not have an induction process for new coaches, I recalled this as something the overall club wanted to work on during the CoP.

The individual emails contained the consent form, interview guide, and a request for a convenient date and time to conduct the virtual interviews via Zoom Inc. COVID-19 restrictions, at the time of data collection, meant face to face interviews were not an option. However, coaches were familiar with the platform from the CoP sessions. Synchronous interviewing, like this, may allow participants to be more forthcoming with information and are more time efficient and convenient (Sparkes and Smith, 2014). Conversely, though, the online interviews are sometimes considered impersonal and may lead to missing verbal and non-verbal cues from the interviewee (Sparkes and Smith, 2014); alongside possible technical difficulties for the participants (Archibald et al., 2019). Nevertheless, the advantages of single click access, live transcriptions, password enabled access, the waiting room feature (Lobe et al., 2020), and security and interactivity levels (Archibald et al., 2019) outweighed any disadvantages. These interviews took place in May and June 2021, were conducted at a time convenient to the women, and were voice recorded only, with permission. They were not video recorded, but Zoom's Closed Caption transcription function was enabled, and the participants were shown how to hide it to avoid distraction. This file formed the starting point for the verbatim transcribing of the participants' interviews which ranged from 28 to 90 min in duration with an average of 44 min.

### Participants

Eleven women coaches (club A *n* = 4, club B *n* = 4 club C *n* = 3) were interviewed and all were white, middle class, non-disabled, heterosexual, and Irish. Table 1 outlines the characteristics of the eleven coaches and Table 2 shows background detail on the participating clubs. The interview guide concentrated on their club specific coaching journey. All participants could withdraw at any time, without repercussions, and their names and clubs were given a pseudonym. Keeping the 11 coaches anonymous, was important due to the parochial nature of the sport and the promotion of the NGB led CoP initiative. Anonymity was upheld throughout the analysis and write up phases with names, locations, or any other identifying material removed or changed. Kaiser (2009) believes that anonymity and confidentiality in research, protects participants from harm if they are unidentifiable but stresses that anonymising the data should not lead to the participants' voices being suppressed. Because the use of quotations could lead to the reader deducing who the participants are, this study uses creative non-fiction composite vignettes, which combined the stories of all 11 coaches. Douglas and Carless (2009) support creative non-fiction suggesting it gives a cloak of anonymity to participants. Likewise, Smith (2013) consider such research more accessible to those outside of academia, as the credible characters can resonate with people and promote dialogue. Based on these benefits, creative non-fiction vignettes were employed to represent the findings from the interviews.

**Table 1:** Characteristics of the 11 participant coaches.

**Characteristics**	**Description ranges**
Age (years)	20–52
Parental status	Parents (*n* = 9)
Coaching experience (years)	2–10
Coaching category Level of sport specific coach education	Novice (*n* = 5), Experienced (*n* = 4), Player-coach (*n* = 2) Foundation to Level 1
Team coaching for 2021 season	Under 6 to under 14 age group
Future plans as coaches	Willing to continue with daughter's group (*n* = 5) Would like to progress to older age groups (*n* = 6)

**Table 2:** Characteristics of the three participating clubs.

	**Club A**	**Club B**	**Club C**
Location	Rural	Urban	Rural
No. of playing members	109	60	138
Age groups catered for	Under 8 to Adult	Under 8 to adult	Under 6 to adult
No. of coaches in the club	19	9	20
No. of female coaches (%)	7 (37%)	6 (67%)	3 (15%)

### Interview Guide

The interview guide was informed by the literature and my personal experience (Sparkes and Smith, 2014). Table 3 outlines a sample of the questions and the sources used to form the interview guide, which was emailed to the coaches before they signed the consent form. A pilot interview, with a woman coach, known to me, was completed to test the guide but this data was not included in the analysis phase. Following feedback and reflection, some questions were removed, e.g., outlining what you would do differently when starting as a volunteer. The interviews sought to illicit answers from the women regarding their club specific experiences and the influence of said experiences on their current and future coaching. A sample of questions used to understand their club specific context from their perspective included—What should be done to attract and retain more volunteer women coaches in your club?, What do you think the club currently does well for their volunteers in terms of supporting them?, What areas would you like support in to develop your coaching further?

**Table 3:** Details of the interview guide.

**Topic of interest for interview guide**	**References**	**Examples of questions used in the interview guide**
Motivations for starting coaching	Harvey et al. (2013)	What were your main reasons for getting involved in volunteering with your club?
Obstacles and achievements in coaching and relationships with other coaches	Norman (2010)	Can you recall any positive or negative experiences you had while coaching? How would you describe the relationship with your fellow coaches? How would you rate the collaboration between coaches in your club?
Confidence and autonomy levels in their coaching	Norman (2008)	How confident are you in your coaching ability? How much autonomy have you in your coaching?
Club specific coaching experiences	Shaw and Allen (2009)	What do you think the club currently does well for their volunteers in terms of supporting them? How has your role in the club changed over time? What areas would you like support in to develop your coaching further?
How relevant the CoP was to their respective role	Clements and Morgan (2015)	How relevant is it to your role with the team? What elements would you like more of? Less of?
Support structures as a coach	Hogan et al. (2021)	What should be done to attract and retain more volunteer women coaches in your club? Who do you discuss your volunteering highs and lows with and perhaps seek guidance from and why? How would you describe your relationship with your fellow mentors and players in the teams you work with?
Future coaching plans	Vinson et al. (2016)	What are your future LGFA coaching plans? What motivates you to continue volunteering?

I continuously recorded notes to a self-reflection journal both during and after the interviews. During the CoP, I noted interactions, as suggested by Sparkes and Smith (2014), to identify what is not happening as much as what is happening and look for recurring patterns, actions (saying and doing), and reactions (responses to what is said and done by others). These field notes helped to inform subsequent discussions (Jones et al., 2012) during the interviews and the analysis phase.

### Analysis

**Figure 1:**
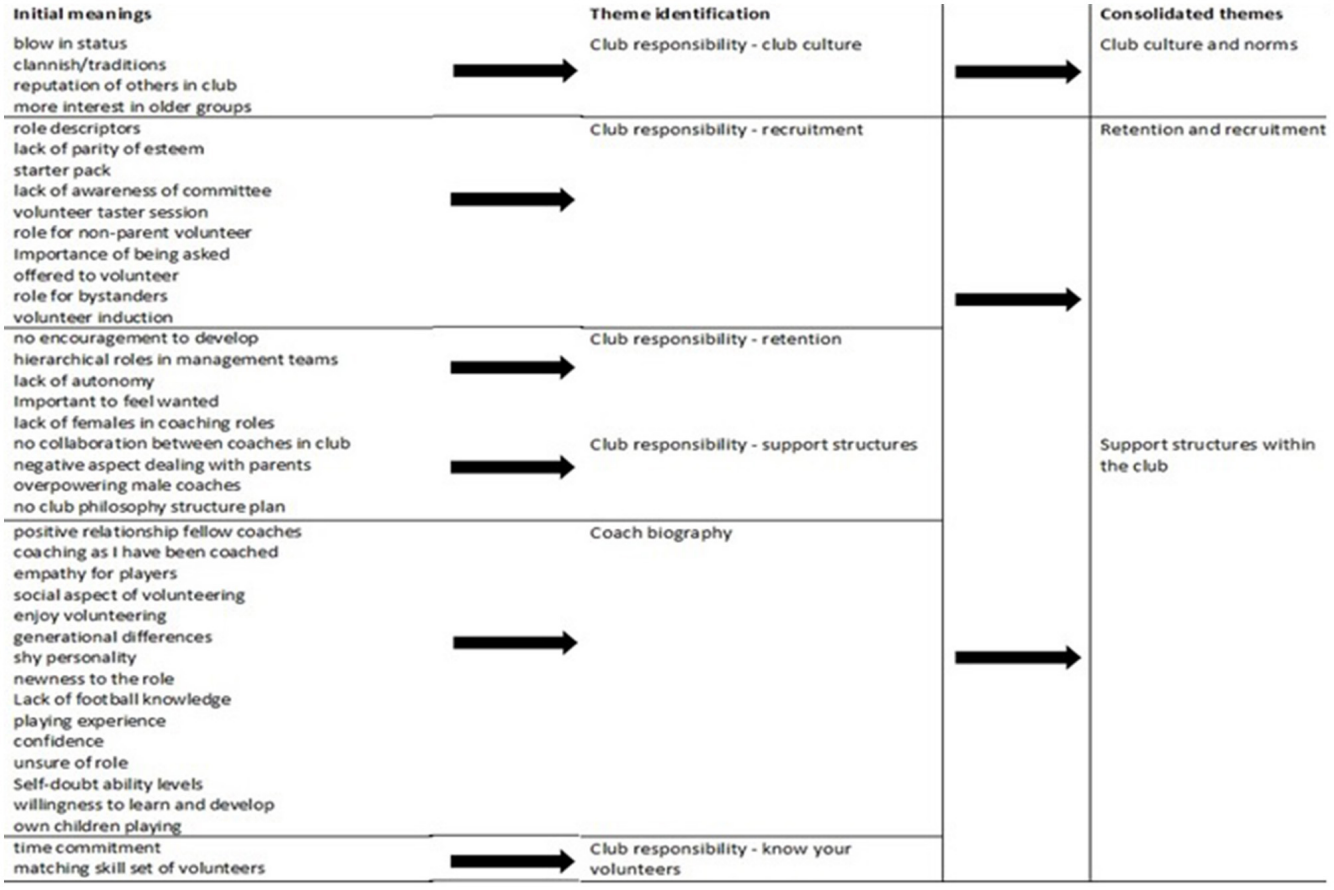
Transition from 40 meanings to consolidated themes.

The data analysis was managed using the N-VIVO software package (Version 12) and occurred in two stages. Firstly, the interview transcripts were inductively analyzed using Braun et al. (2016) reflexive thematic analysis (TA), which then led to the creation of three coach profiles with interwoven themes. The six-step reflexive TA was followed through constant engagement (Braun et al., 2016) with familiarization and coding (phase 1-2), which occurred naturally while conducting and transcribing the interviews. Theme development, refining, and naming (phase 3-5) were influenced by my women's Gaelic football experiences. Forty initial meanings were extracted, usually in the coaches' own words to keep the story close to their lived experiences (Carless et al., 2014). These meanings were consolidated into six themes that included recruitment, retention, knowing your volunteers, support structures, club culture, and coach biography. Figure 1 shows the transition from initial meanings to the consolidated themes. The sixth step of the reflective analysis was undertaken in writing this manuscript and the overall doctoral thesis.

The second stage of creating the composite vignettes was to resonate with the readers and showcase the unique profiles and perspectives of the coaches, while not representing any one coach, but instead displaying the commonality of the identified themes (Paquette et al., 2019). The Smith et al. (2015) study on creative non-fiction supported the process of developing the vignettes. In the context of this study, the interviews were the predominant source of data collection. Creating the vignettes was challenging and there was a process undertaken, firstly through assigning the 40 meanings to the three coach profiles. Stride et al. (2020) seek research to understand the lived experiences of volunteer women coaches through showcasing their differences, hence this study views three different types of volunteers; albeit all are white and able bodied. Nonetheless, it was deemed that these profiles were a mix of the 11 coaches and are reflective of the types of women coaches active in women's Gaelic football clubs. I am not proposing that the scenes or the conversations that are depicted in this transcript are the actual events that took place (Lewis et al., 2020) or that the stories told happened in that exact order for the coaches but instead these are told to best reflect the themes (Smith, 2013). I have used the interview data along with my observations and lived experiences to best describe the key themes that I selected to answer the research question. The penultimate step was to give the profiles a name and make them as real to life as possible to stir reflections (Paquette et al., 2019) and an interpretive response from the reader (McNeill et al., 2017) in relation to the themes. The construction of characters centered on who they were, what story they would tell, and how they would interact with each other, through the use of dialogue, to bring the characters and the story to life (Smith et al., 2015).

Trustworthiness and credibility can be gleaned from the fact that I outlined my background as a coach and coach developer with years of experience in a similar club setting and the second and third authors acted as “critical friends” similar to studies by Smith (2013) and Cavallerio et al. (2017). The second and third authors are involved in women's Gaelic football clubs and one is a female volunteer coach that could resonate with the stories and appreciate them from the participant's viewpoint. Similar to Smith (2013), the critical friend's approach was used to check for evocation and cohesion in the stories. As critical friends, they questioned if the content was written in the participants' words or more formal and reflective of my researcher tone. For example, initially, the vignettes included terms like “club philosophy” and “matching volunteer skill-sets” which were later removed as those terms were not used by the women. Furthermore, trustworthiness was considered as the vignettes and letter were sent to all participants to garner if they were reflective of their lived experiences and how the letter might be used in their club, if at all. This process clarified the usefulness of the club letter, and the compatibility, with elements of the coach profiles and stories but did not lead to any requested changes to the vignettes. This approach was in line with Smith and McGannon (2018), as not considering it as member checking to prove the rigor of the study but instead as a form of member reflection to add to the research.

The plot and story line needed to have a start middle and end through the use of scene setting and internal dialogue (Smith et al., 2015). The vignettes were constructed to tell the important points as opposed to all of the themes being included in each story (Smith et al., 2015). Therefore, any miscellaneous themes were removed or consolidated e.g., knowing your volunteers was subsumed into the support structures in the club, and retention and recruitment were combined as a single theme. The themes are evident from all three coaches' stories suggesting that while one might be more prominent for one coach they are relevant to all participants (Clarkson et al., 2019). The three profiles are reflective of the 11 coaches interviewed and show three typical stages of a coaching pathway; one with long term experience, one novice coach and one that both plays and coaches for the club. All 11 women coaches are categorized into novice, experienced or player-coach, for the purpose of this study with some of their experiences and direct quotes reflected across the three vignettes. Finally, once the vignettes were created, I read and identified their central messages to determine if they matched the main themes and kept in line with the coaches spoken word as reflected in the Results section.

## Results

As involvement in the CoP was publicized locally and there were only a few women active in the process, anonymity was a concern. Therefore, the results compiled in the composite vignettes include the key themes across the interviews, as told from three perspectives. These vignettes are intended to illustrate the responsibility on clubs to provide an appropriate environment for the support and development of women coaches. The vignettes are a novel way to present the findings; yet, it was not the intention during data collection to create these coach profiles to tell their story, but it is the form the analysis took once themes were identified. The six themes (recruitment, retention, knowing your volunteers, support structures, club culture, and coach biography) were subsumed and then depicted as three themes: (i) retention & recruitment, (ii) support structures in the club, and (iii) club culture and norms. Direct quotes and own words from the coaches are interspersed throughout the vignettes to maintain their voice (Clarkson et al., 2019). The coach stories are based on three distinct female coaches with various backgrounds and motivations for starting and staying in coaching and are representative of the eleven coaches in the study.

Amy is a novice volunteer coach and the vignette shares her early experiences, particularly on the first night and 2 months into the role. Brenda reflects on her 8 years of coaching involvement in the club, before sharing her experiences with her friend that is interested in volunteering. Carol is the third coach, who has a few years of coaching experience and also plays with the adult club team. It is hoped the reader will identify the three key themes intertwined in the three coach profiles. The final part of the results section is a letter drafted to the club on behalf of volunteer women coaches, with recommendations of how to enhance their experiences, based on the core themes.

Coach 1, Amy reflecting on her first night as a volunteer coach.

It is my first night at training and it is also the first night of the new season. Before this, I was a bystander parent watching from the side lines, and just standing around, but I was never asked to assist until recently when John asked me. I am glad he did, as I could not say no in person, even after ignoring the numerous texts requesting parental involvement. The personal touch of being asked to help was the only approach for me, as I would not have the confidence to offer my assistance otherwise. Also, the timing is better now as my children are older and I have more time to give back to the club. I am apprehensive though as I have no confidence in my coaching ability and no previous playing experience, but I really want to be involved with the team, particularly as long as my daughter is playing with them. As a result of these nerves, I arrive early for the session. Driving to the pitch I think about what would make tonight's session easier for me and other coaches starting on their journey, such as a session plan and knowing what is expected of me. Perhaps a taster session would have helped, with an experienced coach describing the role and setting out drills to show us what works for different age groups. I then recall the reasons why I decided not to volunteer in the past, which were all focused on barriers I perceived regarding the key people in the club. These barriers included whether you have a child playing, are from the locality, and/or have previous playing or coaching experience.

I refocus on the present and think about the upcoming training session. The butterflies in my stomach notch up a few gears as I wait patiently at the entrance to the pitch for John, the main coach, to notice me. The minutes go by as I observe John chatting with the other two male coaches, Mike and Barry. Mike does not have a daughter playing with the team, but he is an ex-player and highly regarded as a knowledgeable coach in the club. All three male coaches have been with my daughter's team for the last number of years.

As the coaches move onto the pitch, I approach and greet them and John says, “Oh sorry, I totally forgot you were starting tonight, fair play for coming, what would you like to do?” To which I respond, “Just watch, if that is ok?” And that is how the session transpires, I stand on the outskirts and almost return to my bystander role. The induction training and supports I envisioned, did not materialize.

Two months have passed, and I am still in observation mode. My duties are limited to bathroom visits, tying shoelaces, and liaising with referees. I am eager to learn as a coach, but my biggest fear was not having the knowledge or confidence to demonstrate skills, or plan and deliver a full session. However, I feel I am the token female. Admittedly, I enjoy my involvement, particularly when my daughter is present. I also enjoy getting to know people. as I am a blow-in to the club. I often reflect on my expectations and consider, is this the reality for all female parents/bystanders who start volunteering? I will continue until the end of the season, and although I would love to stay volunteering for as long as possible, I am unsure if I will, in a similar capacity, thereafter. I wonder if the coaches, or indeed the club, will recognize this and support my desire to build confidence and competence as an active coach.

Coach 2, Brenda, reflecting on her coaching journey before chatting to a friend that is considering volunteering.

My friend Debbie wants my opinion on starting to volunteer in our club, causing me to reflect on my experiences to date. I am in my 9^th^ year and I would describe my involvement as at a distant level. I only recently considered myself a coach as opposed to a helper role that has minor status in the club. So why did I not see myself as a coach before this?, Was it because (i) I only got involved when my daughter started playing, (ii) I never played, (iii) I am not from the area, (iv) of my low self-confidence as a coach or (v) the club never recognized me as a coach? My main role was handling the paperwork, while the men did all the coaching and I supervised if the players needed to go to the toilet. I help out when the coaches want me, but normally, I hang back and leave them to do it and they have always been happy to take over. Now that I see myself as a coach (after the CoP facilitator referred to me as one), maybe I need to query why my voice is not heard within my coaching team. Being completely ignored like this has caused frustration and led me to question my future involvement. I really enjoy doing something with my daughter that she likes doing and watching her get better at it. I would like to continue coaching until my daughter gets to the age where they do not allow coaches like me, as it will be too competitive. However, I do not feel encouraged to continue coaching by the club or my fellow coaches. I would have to push myself, probably too much, but I will not be pushed out by other coaches either.

I consider Gaelic Games as an integral part of the community and I enjoy the social aspect of volunteering and getting to know new people, because I am your traditional blow-in. The club prides itself on prioritizing fun and participation although I have never seen or heard anything communicated by the club as to how we should conduct our sessions. We are all left to our own devices and there is little communication between club coaches or even from the club committee, who only get involved at the start of the year, or if there is an issue. It is only since the CoP that I have thought about what our individual skills are and if they are being utilized to the best effect. To me, they are not, and the basic requirement of coaching seems to be having a daughter involved with a team or being an ex-player.

So, what would I like or expect my club to do to support and retain me? My club cannot instill equality for all volunteers in the club and feel on par when the men's club has more participants, facilities, coaches, and finances. Our side of the club is not taken as seriously as the men's, even though we are all representing the same community. Is this our fault? Is it the culture or is it because at a national level they are two distinct organizations and we are considered the second tier? What will change this? I want to continue coaching so I think the club must tackle conscious and unconscious bias in relation to gender, parental status, playing experience, and blow-in status. I think most people do not realize the biases and it is just accepted as the norm. I am now at a crossroads; the team is moving to the under 16 age group next year, which is more competitive, and I do not know how I will fit in. I want to and I would like the club to, ensure I am a retained volunteer and not another lost one.

Coach 3, Carol is an adult team player that also coaches and has been asked to encourage her team mates to start coaching.

A club administrator has asked me to encourage my teammates to start coaching. While my experiences are mainly positive, I have also experienced some negativity. I volunteered as part of my college course as an assistant coach and now I am the main coach with an underage team. I liked being the assistant coach, as it was less pressure to come up with drills and ideas. There is a lack of coaching resources in our club, so I probably coach how I have been coached and get ideas from my coaches, who incidentally have always been male. From early on, I was left on my own to do training, which definitely built my confidence.

My negative experiences have centered on my age as much as my gender as it is mainly male coaches in the club, and all are middle-aged or older and I feel they treat me differently. I have experienced overpowering male coaches within my club and with opposing teams. Sometimes when you go to matches, they tend to liaise with Nathan as the man, even though I organized the game and he is my assistant coach. I feel even the parents treat me differently to Nathan. There is a lack of equality in the club between the boy's and the girl's teams so maybe that is where the different treatment stems from. I find that girls are often shoved aside and men always seem to be the main driving force behind the club or even if the boys won the league and the girls won the league; there would be more thought of the boys winning it.

Things have happened to me as a female that would not if I was a male but if I were ever to stand up and say something, it would become a bigger situation. I believe the club could do more to improve these aspects that may discourage younger female coaches. However, I think the committee avoid confronting people as we cannot afford to lose volunteers. It would also be great if we could get more support from the men's side of the club, as we are sometimes overlooked by the men's committee and coaches are more difficult to get, whereas they will jump for the men's side. It also seems easier to get the parents involved and it is unusual for me to be involved, as I am the only person involved in underage coaching that is not a parent.

Thankfully, the negative confrontations are rare, but I need the club to recognize that I have other commitments outside of football. It is difficult when I am playing and coaching because we have similar seasons where sessions often clash. I need reassurance from my club that the assistance is there for me to miss sessions on such occasions. For my teammates, I think there is also a fear of the unknown and fear of long-term commitment. Maybe if we were allowed to coach with our friends or buddied up with a more experienced coach or take a part of the session that we are comfortable with, then more might start coaching. It would be great if there was an easy route for us as adult players from playing to coaching in the club. Overall, though, I enjoy coaching and I think my peers would too, but we need more work from the club to make this happen.

From gaining an insight into the three coaches' journeys, one must acknowledge the club environment plays an integral role in the volunteer women coaches' experiences. Hence, the following is a letter from the women coaches to their clubs with recommendations based on the significant themes.

Dear Club,

We are writing on behalf of current and future volunteer women coaches in the club with our proposed changes, under the umbrella of retention and recruitment, support structures, and club culture and norms. We highlight some key considerations under each heading that we believe will help all women coaches in this club.

**(i) Retention and Recruitment**

- Approach females in your club such as the bystanders, G4M&O, and current adult players.
- Use your current coaches as recruitment personnel to ask people they know in a more targeted manner
- Conduct a taster and/or induction session for all club positions to show what the role entails
- Devise a starter pack to include:

° A typical session relevant to specific age groups with coaching resources
° Clearly defined role descriptions
° Requirements of new volunteers regarding coaching qualifications, safeguarding, etc.

**(ii) Support Structures Within the Club**

- Appoint a volunteer officer as the connection between the volunteers and the committee who can organize training, support, and collaboration between all club coaches.
- Know your volunteer's biography—What is their background? What are their skills and competencies and match these to the roles required in the club? Use the training needs to create a pathway for all coaches to develop and progress.
- Have regular check-ins with volunteers throughout the season both formally and informally.

**(iii) Club Culture and Norms**

- Introduce a club philosophy, informed by all stakeholders, i.e., committee, parents, coaches, and players, and communicate it to all members. The coaching philosophy should include what is important to the club, e.g., playing experience, coaching qualification, coach-athlete relationships, and characteristics of good coaching.
- Recognize and reduce the club's barriers to participation
- Champion parity of esteem

° between the men's and ladies club in the community, and organize bias awareness training in relation to gender, age, blow-in status, playing experience and parental status
° that coaches and helpers at all levels are valued equally in the club

- Challenge those that are not conforming with the club philosophy

While we appreciate the above will take time to implement, we are confident that they are achievable in the short-term, leading to positive long-term benefits for all involved.

Yours Sincerely,

Current and future women coaches.

## Discussion

For both anonymity purposes and in order to communicate the women coaches' voices, creative non-fiction was chosen to present the findings. The organizational layer of the Ecological Intersectional Model (EIM) (LaVoi, 2016) and the Psychological Contract (PC) were the foundations for the design and analysis of this research. This study extends previous research by illuminating the influence of the non-elite club environment on the experiences of volunteer women coaches and provides a starting point for clubs to examine their organization. The vignettes illustrate the experiences of three coaches within the same sport but at different stages of their coaching journey through three different profiles. The first profile was a novice coach, the second an experienced coach, and the third, a player-coach. These three profiles are representative of all 11 women coaches interviewed. Each story portrays individual lived experiences and overall themes common between them, namely retention and recruitment, club culture and norms, and support structures within the club. The stories also highlight the recommendations for clubs to address some of the challenges they faced and further enhance the positive experiences mentioned, starting with retention and recruitment.

### Recruitment and Retention

Internationally volunteer coach recruitment and retention are issues most sports systems have to overcome (Hoye et al., 2019). Recruitment and retention are closely linked and this study argues for increasing the effort in retaining current volunteers to reduce the need for continuously seeking new ones. Nagel et al. (2019) concur and propose that volunteer retention should be a focus, along with recognition and support, in the form of splitting workload among volunteers. Sharing the workload and not expecting too much from a small number of volunteers is a priority for women coaches that often have greater caring roles and responsibilities within the home. Plausibly, the profiles created in this study, namely a novice coach, an existing coach, and a coach that is still playing, are representative of female coaches in most women's Gaelic football clubs. Due to the parochial nature of women's Gaelic football, people rarely move to a different club, unless they are moving residence. Yet, they may volunteer their time with other non-profit organizations instead or quit. In this regard, it is essential that clubs know their volunteers and their respective needs, in order to establish the necessary support.

Rundle-Thiele and Auld (2009) argued that several factors could lead to someone quitting coaching. These include burnout, concerns about self-efficacy, lack of enjoyment and lack of time. Contrary to some narratives that confidence is not a barrier for women coaches, Clarkson et al. (2019) argue that it is; especially at non-elite and recreational levels, and this study supports these findings. The coaches refer to a lack of confidence in their coaching ability and allude to ways it could be improved by the club, which is positive, given that Robertson (2016) state low confidence contributes to the under representation of women coaches. Moreover, the clubs in this study have women coaches at underage but their potential fallout from coaching roles with older age groups, due to a lack of confidence or not seeing a place for them, is concerning. Research like this study is similar to that proposed by Rundle-Thiele and Auld (2009) which suggests that more focus is needed on both the pull and push factors that impact coaches' decisions to stay or leave the organization.

Females coached by females are more likely to stay in coaching (Wasend and LaVoi, 2019). This is a pull factor that bodes well for women's Gaelic football clubs, as if current players are coached by females, they too might transition into coaching. Along with seeing female coaches, an opportunity to sample coaching in a supportive environment with their peers can help females make an informed decision on coaching, as opposed to basing the choice on stereotypes that do not view women coaches as the norm (Soler et al., 2021). Carol, as the player-coach, advocated for this approach to encourage her team mates to start coaching by allowing them to work together with support from a more experienced coach. Moreover, playing experience is crucial for women coaches' sport specific knowledge and confidence levels (Allen and Reid, 2019) and is heavily linked to women starting coaching (Wells, 2016). Amy and Brenda consider their lack of playing experience as a weakness and refer to their fellow male coaches' extensive playing experience equating to a reputation as knowledgeable coaches. This thought process is reinforced by a Gaelic Games coaching survey where almost 20% of the female coaches have never played and only 30% playing until adult grade in comparison to 4% and 58% respectively among male respondents (Horgan et al., 2021). In fact, similar to Carol, 29% of females in the same survey were still playing while coaching, in comparison to 19% of their male counterparts (Horgan et al., 2021). This further highlights the throughput current players might be as future coaches. Recruiting players is advantageous, as they are familiar with the organization compared to those external and without previous involvement; whom require different recruitment practices (Hoye et al., 2019).

Although the coaches' experiences varied, they are eager to stay involved with their respective coaching roles and need the club to do more to ensure their retention. Volunteers are more likely to stay if they feel needed and attached to the club; even if overall job satisfaction is low (Schlesinger et al., 2013). This study is in accordance with the findings of recent studies that claim retention of women coaches is also connected to the involvement of their children (Schlesinger et al., 2013), as mothers will often start coaching coinciding with their child's involvement (Leberman and LaVoi, 2011) and will continue as long as they stay involved (Busser and Carruthers, 2010). Thus, highlighting the importance of player retention for this purpose and also as Clarkson et al. (2019) reported, players can be a group of potential coaches. However, in order to fully support female players transitioning into coaching, it is important that they are encouraged and supported from the beginning of their journey.

### Support Structures Within the Club

This study, along with Clarkson et al. (2019), suggest that support structures and networks are important components in retaining volunteer women coaches. To establish appropriate support structures, it is imperative that clubs know their volunteers' biographies, so they can meet their expectations through matching their skill-sets to suitable roles. Egli et al. (2014) recommend the use of an entry questionnaire or interview, to assist with meeting expectations. However, this study proposes a less formal introductory process through inductions and asking people to get involved, which Amy promoted, over generic calls for parental assistance. This informal, but individual, approach will aid open communication and provide information regarding the club goals (Schlesinger and Weigelt-Schlesinger, 2013), which Brenda calls for, as she is not aware of any communications about her club's aims, even after being involved for 8 years.

Although volunteer coaches are time poor (Walsh, 2015) they are still willing to learn and develop to gain confidence and competence in the role (Walsh et al., 2011). While the majority of LGFA qualified coaches at the introductory level are female, this figure may be skewed as the sport is relatively new, and historically, coaches completed the male sport equivalent course which is recognized as suitably qualified. Furthermore, it is known, anecdotally at least, that the number of active volunteer women coaches is not reflective of this statistic and so data regarding the development and retention of women coaches is warranted. This study outlines the experiences of the women coaches and while they have been recruited successfully, Amy, Brenda and Carol may leave coaching, if the correct structures are not implemented to support and encourage them to progress; particularly as their daughters progress to the older age groups. Hancock et al. (2017) refer to the “leaky pipeline” for female administrators in collegiate sport and Wells (2016) refers to it with females as professional coaches, so the same could be true for volunteer coaches progressing through the age groups. Females need role models to see coaching as an active possibility (Schull, 2016), so ensuring effective support structures are in place has the potential for a snowball effect, which could lead to the recruitment of more women coaches.

The coaches suggested favorable recruitment practices should include taster sessions, guidance from more experienced volunteers, and a point of contact, such as a volunteer officer. Matching the needs and expectations of volunteers is crucial to their longevity and commitment to the role (Egli et al., 2014). Having an organized method of matching experiences and meeting expectations could be within the remit of a volunteer officer. Hogan et al. (2021) propose a volunteer role at the administration level in clubs and this study furthers the proposal by outlining some of their duties. Such functions could include a point of contact between the committee and coaches, along with facilitating inductions and developing a starter pack for new volunteers. Amy, reflecting on her first night of coaching, endorses some form of induction and a starter pack and Brenda supports this further by alluding to the lack of matching volunteer skill sets. From Carol's perspective, there is a lack of coaching resources in her club, which causes her to coach how she has been coached. Early stage assistance should lead to a positive experience for women coaches, and further club-based support structures are essential throughout their coaching journey.

The club can provide club based training and as with Søvik et al. (2017), a move away from traditional coach education to collaboration among coaches of different levels is proposed. Engaging in coaching dialogue will connect volunteers (Allen and Reid, 2019), particularly if coach development focuses on a common goal (Clements and Morgan, 2015) through learner centered content, based on the coaches' biographies (Leduc et al., 2012). The coaches' biographies can be used as a starting point to recognize their training needs and create a pathway for them to develop and progress. However, to react to the coaches' needs, based on their biographies, there is an onus on clubs to know their volunteers and understand their background, strengths, and areas for development.

A CoP could suit coach development, where ideas and expertise are shared in a safe environment. The success of a CoP will depend on a dedicated facilitator, regular meetings, common goals, and building on coaches' strengths (Bertram et al., 2016). Although Bertram et al. (2016) believe coaches can set up their own CoP, the findings of this study indicate, a clear readiness for change and acceptance of a move away from the traditional coach education, as prerequisites. Furthermore, as indicated by Brenda, club coaches operate in isolation with their particular age group, which was deemed a negative aspect of her club. This lack of formal or informal collaboration between club coaches needs addressing, in the first instance, to reap the benefits of working together. Once collaborating, volunteers use their networks with fellow coaches for guidance, conflict resolution, mentoring (Harman and Doherty, 2014), and solving coaching problems (North et al., 2020), which can then become the norm. These progressive and combined approaches of coach development could be introduced in the club and if established may lead to a positive club culture relating to coach development. These measures align with Rundle-Thiele and Auld (2009) in that the decision of volunteers to stay or leave is predominantly a personal one. Yet, the organization can impact the decision by ensuring a positive environment and culture for volunteer coaches. Importantly, Millar and Doherty (2018) claim that readiness to change is a significant factor in whether or not community sports organizations can build their volunteering capacity. This study shows organizations the starting point for their respective clubs so that they can first identify what needs changing and then address it, which may include the club culture and norms.

### Club Culture and Norms

The norms and culture in a club can have both a positive and negative impact on the women coaches' experiences. Hogan et al. (2021) call for women to be viewed as more than female liaison personnel and to recognize them as coaches, which may require club members to reduce their biases through awareness training. Carol, as the youngest of the three coaches, experienced gender and age-related bias, as she felt the older male coaches treated her differently. Yet, the male coaches were never confronted by the club, in case they stopped volunteering. There is a need to challenge such behavior through awareness training on how this could negatively impact the experiences of the younger female coaches. This is supported by Walker and Sartore-Baldwin (2013) who imply that societal views need to change to deem women coaches equal to their male counterparts, as typical social norms often prevent opportunities for women in coaching.

All three women coaches profiled alluded to a lack of equality between the women's and men's side of the club. Similar to Clarkson et al. (2019), this study supports the finding that women coaches feel a lack of respect, among their male peers in the club, and women predominantly coach at lower levels of competition. Such conscious and unconscious biases can feed into the culture and norms in clubs. Lewis et al. (2018) purported that women coaches often feel intimidated and encounter sexist comments when in male dominated coaching environments. Addressing such organizational biases includes exploring how coaches conform to these identities to fit into the environment (Burton and Newton, 2021) and clubs could try and disrupt negative discourse and norms for women coaches (Stride et al., 2020). Managing the culture in the sports organization in the form of ongoing communications, philosophy on winning and volunteer coaches feeling valued, are key components (Burton and Newton, 2021). Allen and Shaw (2013) suggest leaders and decision makers reflect on the values, structures, and supports of their respective organizations and memberships.

The culture and perception of considering men to be better coaches than women was apparent in all the coaches' stories and often occurred naturally and without evidence, which aligns with the findings of Schull and Kihl (2019). Invariably, all eleven coaches referred, to their blow-in or outsider status, due to the parochial nature of the sport, which is attributed, anecdotally at least, to the overall Gaelic games culture. While it is regularly used in jest it can also lead to unconscious bias in the club and potentially inflates the status of some coaches, most often males, that have a perceived greater knowledge based solely on their playing careers. This study showed that level of coaching, age, and gender were among the biases that the coaches encountered in their clubs. Additionally, non-parent, women coaches should not be treated as if they have more time or availability to coach, as doing so is also forming a bias (Burton and Newton, 2021). In this regard, reducing the biases that exist within the club on parental status, age, gender, blow-in status and playing experience, will enhance the experiences for existing and future women coaches. It is important for clubs to recognize and address the barriers to participation in their club and they must champion parity of esteem for all in women's Gaelic football.

While the recommendations in this text are considered to have the potential to improve the experiences for all volunteers in the club, one must be cognizant of the limitations facing clubs. Restricted resources in terms of personnel that are often engaged in multiple volunteer roles; is one, along with a collective willingness for change. In this regard, the NGB could consider these recommendations and put the supports in place to train and develop club members to adopt these roles and become more diligent in supporting and developing volunteer coaches at non-elite level. The role of the volunteer officer needs an official title and role description from the NGB, perhaps after trialing it in a sample of clubs over the 2022 season. This would then help inform best practice for additional clubs in 2023. This study advocates an ideal candidate as someone with volunteer coach experience, good interpersonal skills, and an in-depth understanding of the organizational functions within a club.

Further to the organizational level improvements that clubs can make to improve their volunteers' experiences, there is a need to address the culture within the club. The three coaches highlight the cultural imbalance and norms within the club and it is clear that parity of esteem is a difficult ask, and, one that will take time to improve. The women want to feel as valued and important as the men, which requires open communication between both committees, as well as some common activities and initiatives. Those in decision making positions will need to promote this change and invest time, and perhaps, financial resources to have it as a future vision for the club. Ultimately, all the blame should not be aimed at the males but, instead, the decision making positions need to take ownership of what they can do to attract, develop, empower and retain, volunteer women coaches.

In the first instance the culture within the club regarding the role of the female coach and the perception of what their duties should include needs to be addressed. Although Brenda's involvement is lengthy, she continued doing minor tasks and was rarely involved in the coaching aspects. Through her story, it is apparent that the male coaches she has worked alongside take over and she does not feel she has an opportunity to be involved as her daughter progresses to more competitive age groups. This transition from participation to competitive levels suggests that the male coach is the best placed coach in this scenario. It appears acceptable for women coaches to stay with the younger teams, but they do not feel competent, or indeed, welcome with the more competitive teams. There is an obvious power dynamic within the club that, from Brenda's perspective, is heavily based on masculinity, while Carol, a much younger coach, with more playing than coaching experience, has negative experiences from domineering male coaches. While many of the coaches in this study had negative experiences, there was still a sense of overall enjoyment in their volunteer roles. The eleven participants were eager to stay involved in coaching, yet their continuation will be dictated by others in the club. Therefore, it is incumbent on the club to ensure that enjoyment is the overarching sentiment of volunteer women coaches and through adopting some of this study's recommendations, that could be achievable.

While this study focuses on the impact of the organizational level on the coaches experiences, this is only one aspect of their lives and as Potrac et al. (2016) acknowledge, there are various interconnections, social networks, and personal identities that community based coaches must also manage. Hence, this study does not suggest that this is an exhaustive understanding of the women coaches' perspectives in respect of their organizations, as it is complex, with multi-layers and variables impacting their experiences. Volunteer women coaches have varied motivations for coaching, individual challenges, and plans to continue. Therefore, clubs and NGBs must be cognizant of this and try to understand and care about their circumstances (Stride et al., 2020). Furthermore, volunteer management programmes can aid recruitment, development, and retention of volunteers through role descriptions, induction and balancing the needs of the club and the volunteers by considering their perspectives (Hoye et al., 2019), which this study also supports.

## Conclusion

Volunteer women coaches have been under represented in coaching research to date (Pfister and Norman, 2017), even though there has been many policies and initiatives focusing on increasing the number of women in coaching (Harman and Doherty, 2014). This study set out to develop a better understanding of how the club's environment influences the support and development of volunteer women Gaelic football coaches. It is envisaged that this research will resonate with those involved in women's Gaelic football and start a conversation on the role of volunteer women coaches and, more importantly, what is required, from a club perspective, for their support and development. This study purports that these findings are common in many Gaelic games clubs but may also be true for other volunteer sports. Ultimately, knowing and understanding your volunteer, providing the correct support structures, and tackling the negative aspects of the club culture, can lead to positive recruitment and retention of volunteer women coaches. Retaining women coaches is a priority, as women tend to quit volunteering quicker than their male counterparts (Burton and Newton, 2021). While this study focused on women coaches, there is nothing to suggest that these improved organizational practices would not also benefit male volunteers in a non-elite club setting.

Clubs are advised to incorporate some of the key findings from this study to best serve their current and potential women coaches. Accordingly, clubs can use these vignettes as a starting point to identify which category of coaches they have and if the stories are reflective of their lived experiences. Once armed with this information, clubs can plan and prepare their organization in the most context-specific way to retain their volunteers. In the short term, the three clubs represented will have access to the findings to highlight their strengths and possible areas for improvement. More broadly, the LGFA can use the findings to share with clubs to self-assess regarding their volunteer profiles and experiences. Such widespread dissemination may inform current and future practices at the non-elite club level. Within these practices, the role of the volunteer officer is key to the success of any future recruitment and retention initiatives that a club introduces. Training and support for these officers will be necessary from the NGB, in the first instance, as well as support from all club members. This study recommends that clubs devise a philosophy, created by all relevant stakeholders, and anyone in breach of these guiding principles is encouraged to upskill and develop. This research could be used by other NGBs as a starting point to understand the gendered perceptions of their volunteers and use it to build a more positive environment, with a cumulative effect on volunteer retention and recruitment.

The limitations of this study help to focus researchers for future studies such as the lack of intersectional identity of the women coaches; albeit they are representative of the active women coaches in women's Gaelic football in Ireland. Further, the participants were all active volunteers and were interested in continuing, so were presumably having an overall positive experience in their respective organizations. Future research could focus on a more diverse range of female volunteer coaches in the club, to give a voice to those that are starting or have recently left or even those that have not commenced. Additionally, research could focus on the organizational voice when it comes to the support and development of women coaches to see how the two perspectives align. A further limitation is that the three clubs had volunteered for the CoP, indicating their ambition, which may skew the results in a positive way; a less progressive club may unearth an interesting contrast to this study. As such other stakeholders, such as players, parents, and club officers, within the clubs could also be the focus of future research in order to determine their context specific club experiences. Further studies could build on this work and oversee a club that has implemented some of these proposals and determine if volunteer women coaches' experiences have been enhanced as a result.

The use of vignettes and the club letter is a novel approach to showcase research on women in coaching and addresses the opportunity proposed by Smith (2013) to have research available to those outside of academia. The member reflections, of the vignette and club letter, position this study beyond research. They can be used as a tool to ignite discussions and a club audit to establish their status, and potential improvements, in relation to their volunteers. Having the stories based on others may foster a deeper understanding of hidden messages and act as a call to action for changes in the current approaches to recruitment, retention, support, and development of volunteer women coaches at non-elite level across all sports; not just in women's Gaelic football.

## Data Availability Statement

The raw data supporting the conclusions of this article will be made available by the authors, without undue reservation.

## Ethics Statement

The studies involving human participants were reviewed and approved by Mary Immaculate College Research Ethics Committee (A18-035). The participants provided their written informed consent to participate in this study.

## Author Contributions

All authors listed have made a substantial, direct, and intellectual contribution to the work and approved it for publication.

## Funding

Research funding from Sports Ireland Dormant accounts that supported the CoP initiative *via* the Ladies Gaelic Football Association (LGFA).

## Conflict of Interest

The authors declare that the research was conducted in the absence of any commercial or financial relationships that could be construed as a potential conflict of interest.

## Publisher's Note

All claims expressed in this article are solely those of the authors and do not necessarily represent those of their affiliated organizations, or those of the publisher, the editors and the reviewers. Any product that may be evaluated in this article, or claim that may be made by its manufacturer, is not guaranteed or endorsed by the publisher.
